# Dummy run for planning of isotoxic dose-escalated radiation therapy for glioblastoma used in the PRIDE trial (NOA-28; ARO-2024-01; AG-NRO-06)

**DOI:** 10.1016/j.ctro.2024.100790

**Published:** 2024-05-04

**Authors:** Sebastian H. Maier, Stephan Schönecker, Vasiliki Anagnostatou, Sylvia Garny, Alexander Nitschmann, Daniel F. Fleischmann, Marcel Büttner, David Kaul, Detlef Imhoff, Emmanouil Fokas, Clemens Seidel, Peter Hau, Oliver Kölbl, Ilinca Popp, Anca-Ligia Grosu, Jan Haussmann, Wilfried Budach, Eren Celik, Klaus-Henning Kahl, Elgin Hoffmann, Ghazaleh Tabatabai, Frank Paulsen, Adrien Holzgreve, Nathalie L. Albert, Ulrich Mansmann, Stefanie Corradini, Claus Belka, Maximilian Niyazi, Raphael Bodensohn

**Affiliations:** a Department of Radiation Oncology, University Hospital, LMU Munich, Munich, Germany; b Bavarian Cancer Research Center (BZKF), Munich, Germany; c German Cancer Consortium (DKTK), partner site Munich, a partnership between DKFZ and LMU University Hospital, Munich, Germany; d German Cancer Research Center (DKFZ), Heidelberg, Germany; e Department of Radiation Oncology, University Hospital Tübingen, Tübingen, Germany; f Department of Radiation Oncology and Radiotherapy, Charité-Universitätsmedizin Berlin (Corporate Member of Freie Universität Berlin, Humboldt-Universität zu Berlin, and Berlin Institute of Health), Berlin, Germany; g Department of Radiotherapy of Oncology, University of Frankfurt, Frankfurt, Germany; h Department of Radiation Oncology, CyberKnife and Radiation Therapy, Faculty of Medicine and University Hospital of Cologne, University of Cologne, Cologne, Germany; i Department of Radiation Oncology, University Hospital Leipzig, University of Leipzig, Leipzig, Germany; j Department of Neurology and Wilhelm Sander-NeuroOncology Unit, Regensburg University Hospital, Regensburg, Germany; k Department of Radiotherapy, University Medical Center Regensburg, Regensburg, Germany; l Department of Radiation Oncology, University of Freiburg Faculty of Medicine, Freiburg, Germany; m Department of Radiation Oncology, Medical Faculty and University Hospital Düsseldorf, Heinrich Heine University, Düsseldorf, Germany; n Dept. of Radiation Oncology, Faculty of Medicine and University Hospital Ruhr-University Bochum, Marien Hospital Herne, Herne, Germany; o Department of Radiooncology, University Hospital Augsburg, Augsburg, Germany; p Department of Neurology and Interdisciplinary Neuro-Oncology, University Hospital Tübingen, Hertie Institute for Clinical Brain Research, Tübingen, Germany; q Center for Neuro-Oncology, Comprehensive Cancer Center Tübingen-Stuttgart, University Hospital Tübingen, Tübingen, Germany; r Department of Nuclear Medicine, University Hospital, LMU Munich, Munich, Germany; s Ahmanson Translational Theranostics Division, David Geffen School of Medicine, University of California Los Angeles (UCLA), Los Angeles, USA; t Institute for Medical Information Processing, Biometry and Epidemiology, Faculty of Medicine, LMU Munich, Munich, Germany; u German Cancer Consortium (DKTK), partner site Tübingen, a partnership between DKFZ and University Hospital Tübingen, Tübingen, Germany

**Keywords:** Glioblastoma, Dose Escalation, PRIDE Trial, Bevacizumab, FET PET, QA

## Abstract

**Background:**

The PRIDE trial (NOA-28; ARO-2024-01; AG-NRO-06; NCT05871021) is designed to determine whether a dose escalation with 75.0 Gy in 30 fractions can enhance the median overall survival (OS) in patients with methylguanine methyltransferase (MGMT) promotor unmethylated glioblastoma compared to historical median OS rates, while being isotoxic to historical cohorts through the addition of concurrent bevacizumab (BEV). To ensure protocol-compliant irradiation planning with all study centers, a dummy run was planned and the plan quality was evaluated.

**Methods:**

A suitable patient case was selected and the computed tomography (CT), magnetic resonance imaging (MRI) and O-(2-[^18^F]fluoroethyl)-L-tyrosine (FET) positron emission tomography (PET) contours were made available. Participants at the various intended study sites performed radiation planning according to the PRIDE clinical trial protocol. The treatment plans and dose grids were uploaded as Digital Imaging and Communications in Medicine (DICOM) files to a cloud-based platform. Plan quality and protocol adherence were analyzed using a standardized checklist, scorecards and indices such as Dice Score (DSC) and Hausdorff Distance (HD).

**Results:**

Median DSC was 0.89, 0.90, 0.88 for PTV60, PTV60ex (planning target volume receiving 60.0 Gy for the standard and the experimental plan, respectively) and PTV75 (PTV receiving 75.0 Gy in the experimental plan), respectively. Median HD values were 17.0 mm, 13.9 mm and 12.1 mm, respectively. These differences were also evident in the volumes: The PTV60 had a volume range of 219.1–391.3 cc (median: 261.9 cc) for the standard plans, while the PTV75 volumes for the experimental plans ranged from 71.5–142.7 cc (median: 92.3 cc). The structures with the largest deviations in Dice score were the pituitary gland (median 0.37, range 0.00–0.69) and the right lacrimal gland (median 0.59, range 0.42–0.78).

**Conclusions:**

The deviations revealed the necessity of systematic trainings with appropriate feedback before the start of clinical trials in radiation oncology and the constant monitoring of protocol compliance throw-out the study.

**Trial registration:**

NCT05871021

## Highlights

• PRIDE trial aims to improve survival of MGMT-non methylated glioblastoma patients.
• FET-PET supported dose escalation up to 75 Gy is planned.
• This paper analyzes the results of the dummy run of the study centers.
• DICE and Hausdorff analyses show differences, the manual was subsequently improved.
• QA is a central aspect in the planning and conduct of radiation oncology studies.

## 1 Introduction

The PRIDE trial (NOA-28; ARO-2024-01^2^; AG-NRO-06; NCT05871021) combines dose-escalated radiation therapy (RT) with the vascular endothelial growth factor (VEGF) inhibitor bevacizumab (BEV). Its aim is to improve the poor overall survival (OS) of patients with methylguanine methyltransferase (MGMT) non-methylated glioblastoma without increasing toxicity [1]. The poor OS of glioblastoma patients is often attributed to the high rate of recurrences, occurring frequently within the radiation treatment field, suggesting an insufficient irradiation dose for tumor control. Dose escalation can potentially lead to higher local control, while also increasing the risk of radionecrosis [2], [3], [4], [5], [6]. By adding BEV to dose escalated RT, the PRIDE trial aims to counteract the increased radionecrosis risk, thus leading to isotoxicity in comparison with standard treatment [7].

To obtain reliable results in radiation oncology trials, it is important to verify the quality of treatment plans[8], [9], [10], [11]. This includes familiarizing the participating centers with the appropriate protocols and dose specifications. Ohri et al. demonstrated in a meta-analysis that RT protocol deviations occurred in 8 % to 71 % of cases in the examined studies and were associated with an approximately 75 % increase in the risk of treatment failure and overall mortality [10]. Using quality assurance (QA) data from prospective clinical trials, Weber et al. also demonstrated that failing to comply with protocol-specified RT requirements is common [9]. As this not only concerns the successful outcome of clinical trials but also impacts patient safety, optimal QA is absolutely essential. For example, in the recently published SPECTRO-GLIO study, the coordinating center evaluated all contours, including target volumes and organs at risk (OAR), for patients assigned to the treatment arm to prevent deviations [11], [12].

As a first step in our QA concept within the PRIDE trial, we conducted a dummy run asking the study sites to plan and upload one patient case of the recently published test patients [1]. In the subsequent course, continuous uploads of radiation plans and approval from the coordinating center are intended. The present analysis aims to showcase the results of the PRIDE dummy run and illustrate the utility of structured training within the context of a clinical study involving radiation.

## 2 Methods

### 2.1 Dummy run

One benchmarking case containing various study-related challenges was chosen from the test cohort recently published in a separate feasibility study [1]. For comparison with the submitted plans, three radiation oncology specialists delineated all target volumes, coming to a joint agreement on the structure contours. A medical physicist created the plans using Monaco® TPS that uses a Monte Carlo algorithm for dose calculation.

**Fig. 1:**
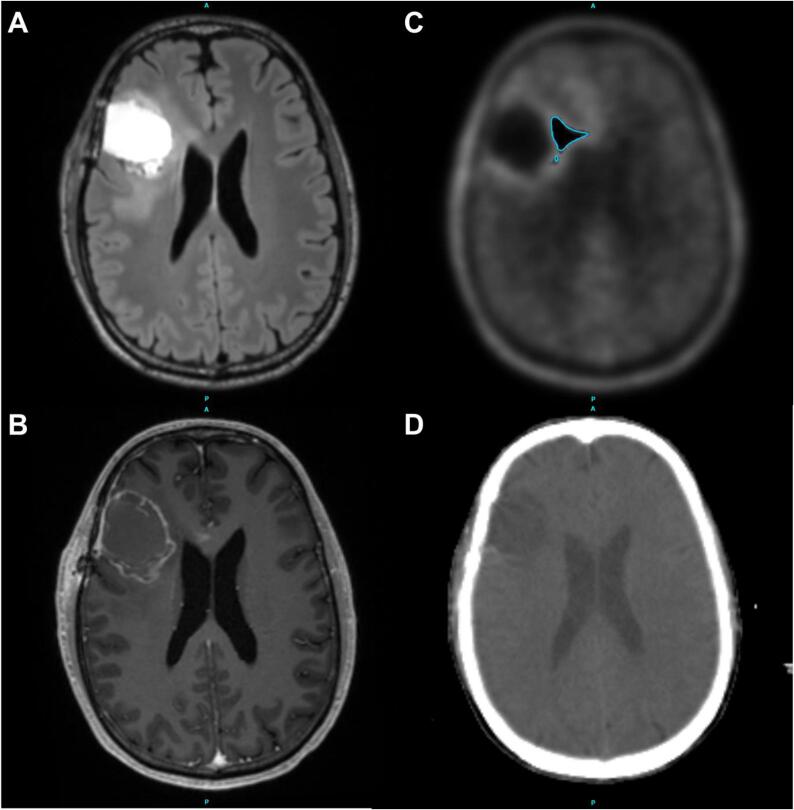
Representative screenshots of the sample patient's images sent to the study sites. A: MRI FLAIR, B: MRI T1, C: FET PET (BTV with TBR of 1.8), D: Planning CT. *(BTV = biological tumor volume; MRI = magnetic resonance imaging; FET = O-(2-[^18^F]fluoroethyl)-L-tyrosine; PET = positron emission tomography; TBR = target-to-background ratio; A = anterior; P = posterior).*

A pre dummy run with one center was conducted prior to the actual run to guarantee accurate technical execution and assess the workflow. This center was excluded from the actual dummy run. For the dummy run, an anonymized Digital Imaging and Communications in Medicine (DICOM) dataset comprising computed tomography (CT), magnetic resonance imaging (MRI, contrast-enhanced T1 and T2 FLAIR weighted sequences) and O-(2-[^18^F]fluoroethyl)-L-tyrosine positron emission tomography (FET PET) scans (Fig. 1) was sent to the participating study sites (n = 8), which are all specialized neurooncological centers, via databox. The FET PET scan included the predetermined structure of the biological tumor volume (BTV), used to construct the unified gross tumor volume (GTVu). Using the target-to-background ratio (TBR) threshold of 1.8, the nuclear medicine physicians assisted in creating the BTV as described [1], based on the FET PET scan. In addition, a manual with the detailed description of the target and risk structure definition as well as instructions for the upload and an anonymization xml.file were provided. The manual contained the recently published adapted PRIDE irradiation specifications [1] incorporating the 2023 European SocieTy for Radiotherapy & Oncology – European Association of Neuro-Oncology (ESTRO-EANO) planning guideline [13]. Each study site downloaded the case into their treatment planning system (TPS) and co-registered the images as per standard contouring procedure. Various TPS were utilized, including Monaco® (Elekta, Stockholm, Sweden), RayStation® (RaySearch Laboratories AB, Stockholm, Sweden), ARIA® (Varian Medical Systems, Palo Alto, California, U.S.), and Pinnacle^3^® (Koninklijke Philips N.V., Amsterdam, Netherlands). According to the manual, a radiation plan for the standard (60.0 Gy) as well as a dose escalated plan (75.0 Gy) had to be prepared, including the relevant OAR. The TG-263 nomenclature has been used to standardize the naming of the OAR [14].The DICOM plans and dose files were then returned to the study center via the ProKnow® platform (Elekta, Stockholm, Sweden). This will also be required for the actual study for each patient. Plan quality and protocol compliance were reviewed and feedback was given.

### 2.2 Plan analysis

After complete upload of the plans, an evaluation of the target volume coverage and compliance with the constraints was performed using the established scorecards according to the protocol specifications [1]. In addition, a calculation of equivalent uniform dose (EUD) [15], [16], [17], [18], [19] and normal tissue complication probability (NTCP) of the brain [20], [21] was performed using the following formula to estimate the respective risk of radionecrosis. The EUD is computed through the formula presented below, considering the irradiated partial volumes (Vi) and doses (Di) in each bin, along with the volume-effect parameter 'a' (where 'a' belongs to the range [1, ∞]. The EUD corresponds to the maximum dose (Dmax) when 'a' approaches infinity, and to the mean dose (Dmean) when 'a' is equal to 1. The study utilized the NTCP model based on the EUD of the brain, which excludes cavernous sinuses, brainstem, optic chiasm, optic nerves, pituitary, mammillary bodies, Meckel’s caves, and GTVu. After the experiences of Niyazi et al., the value of 'a' was set to 9 and converted into 30 fractions [20].

$$\mathrm{EUD}={\left(\sum_{i=0}^{n}{V_{i}D_{i}}^{a}\right)}^{\frac{1}{a}};NTCP=\frac{1}{1+{\left(\frac{55.5Gy}{{\mathrm{EUD}}_{9}}\right)}^{10}}\;$$

As our hypothesis suggests that BEV may reduce the risk of radionecrosis by a factor of 2–3 [1], similar to what has been demonstrated for patients receiving reirradiation [7], our goal is to achieve a similar factor for the ratio of the two NTCP values (NTCP_ref_, NTCP_ex_: the NTCP of the reference and the experimental treatment plan, respectively). A value of 1, 2, or 3 indicates that the risk of radionecrosis is the same, doubled, or tripled, respectively, in the experimental plan compared to the reference plan. A more precise method for assessing the increased risk (IR) of the experimental plan is by using the following logarithmic formula [1]:

$${\mathrm{NTCP}}_{\mathrm{Ratio}}=\frac{{\mathrm{NTCP}}_{\mathrm{ex}}}{{\mathrm{NTCP}}_{\mathrm{ref}}};IR=\frac{\ln(1-{\mathrm{NTCP}}_{\mathrm{ex}})}{\ln(1-{\mathrm{NTCP}}_{\mathrm{ref}})}$$

For an exact comparison of the geometric similarity of the structures, an analysis using Dice similarity coefficient (DSC) and Hausdorff Distance (HD, and 95th percentile HD_95_) was performed to compare the plans from the different study sites with the reference plan. The similarity metric computations were calculated using Plastimatch^3^ [22]. Dice similarity coefficient and Hausdorff distance are well-established volume-based metrics for plan comparison in segmentation studies [23], [24], [25], [26], [27]. DSC characterizes the spatial alignment between two volumetric contours [23]. The DSC, which varies from 0 to 1, quantifies this overlap. A value of 0 signifies no contour alignment, while a value of 1 indicates complete volumetric congruence [24]. HD describes the maximum Euclidean distance between the closest outer surface points for two contours [23], [24]. Due to the sensitivity of the HD to outliers, the 95th percentile was additionally calculated.

$$DSC\left(A,B\right)=\frac{2\left(A\cap B\right)}{A+B}$$

$$HD\left(A,B\right)=\max\left(h\left(A,B\right),h\left(B,A\right)\right)$$

A review process based on the specified parameters was performed by three radiation oncology specialists for all submitted plans, including examining registration, plan quality, and adherence to protocol. Individual feedback was provided, and if deemed necessary, re-planning was requested to address any identified major issues.

## 3 Results

### 3.1 Structure comparisons – Dice Score, Hausdorff Distance

**Fig. 2:**
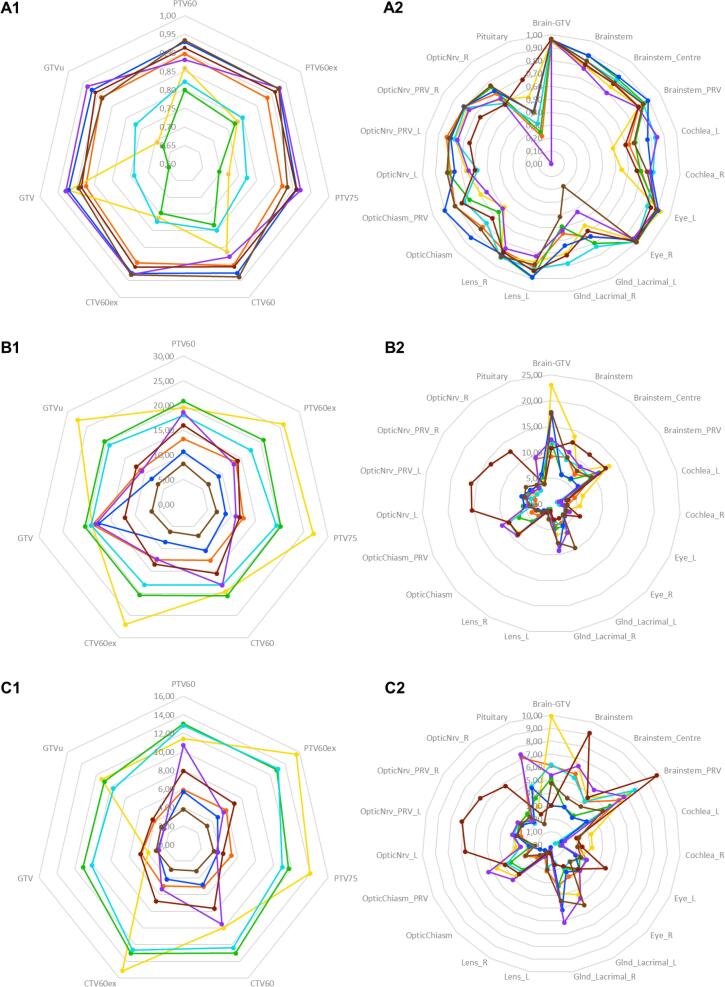
Radar charts for the Dice Score (DSC) (A1 and A2), Hausdorff Distance [mm] (HD) (B1 and B2) and the 95th percentile of Hausdorff Distance [mm] (HD_95_) (C1 and C2); 1 show the target volumes, 2 the organs at risk respectively.

**Fig. 3:**
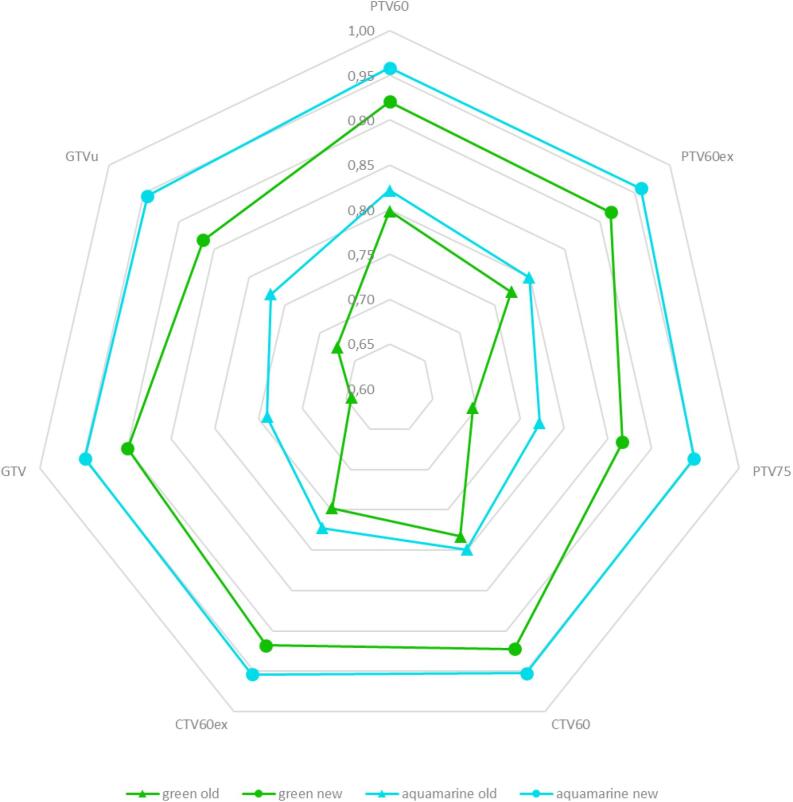
Radar charts for the Dice Score (DSC) for target volumes for the green and aquamarine plan before (old, triangle) and after correction (new, dot). (For interpretation of the references to colour in this figure legend, the reader is referred to the web version of this article.)

**Fig. 4:**
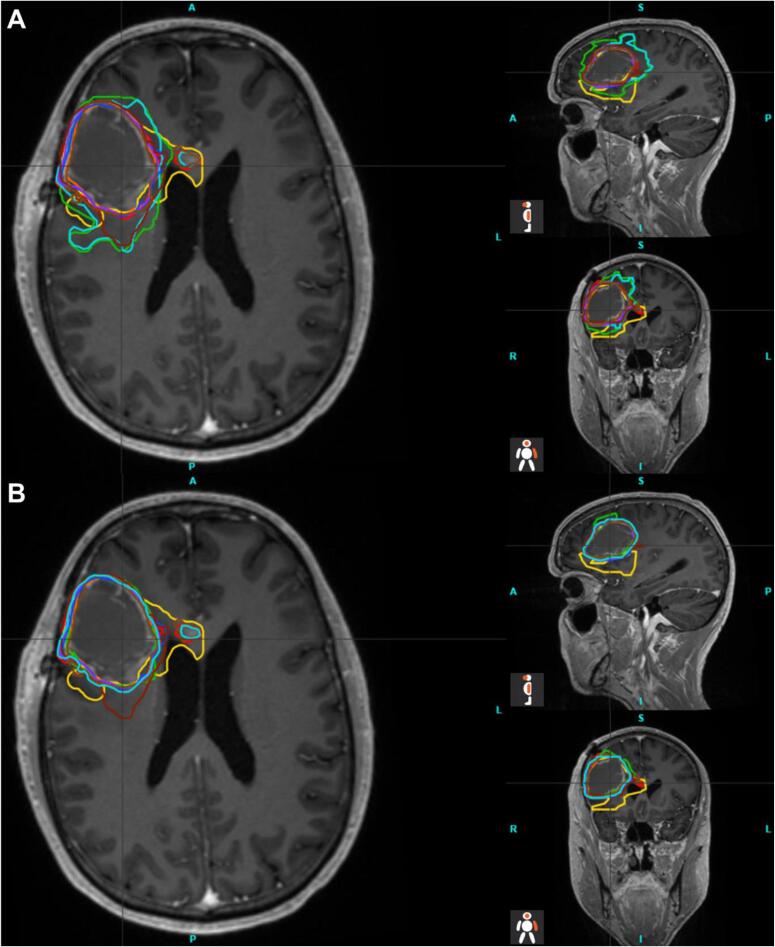
MRI images including the structure sets from all study sites for the GTVu, first round (A) and after correction(B). (*A = anterior; P = posterior; S = superior; I = inferior; L = left; R = right).*

For the majority of specifications, all submitted plans met the specified dose requirements. Nevertheless, there were major differences in the volumes of the target structures (Table 1). Median volume of the planning target volume of the standard plan (PTV60, range) was 261.9 cc (219.1–391.3 cc). For experimental plans, the median volume was 56.68 cc (46.4–102.08 cc) for the GTVu and 92.3 cc (71.5–142.7 cc) for the planning target volume of the 75 Gy simultaneous integrated boost volume (PTV75). In two plans ( green,  aquamarine) the GTV was too large due to the inclusion of the entire FLAIR-enhancement, including huge edema, and thus did not meet the protocol requirements. After clarifying the misunderstanding in the manual, these plans could be accepted in corrected form. The differences can also be demonstrated with the structural comparison parameters (Dice Score (DSC), Hausdorff Distance (HD) and 95th percentile of the Hausdorff Distance (HD_95_), listed in Table 2, and presented in Fig. 2). Fig. 3 illustrates the differences in structures between the center plans for GTVu (A) and after the correction (B) as examples. There are also some major differences in the OARs, especially in small structures such as the pituitary gland. The median DSC is 0.37 (0.00–0.69), the median HD is 5.7 mm (4.0–9.4 mm) and the HD_95_ is 4.2 mm (1.7–7.4 mm). Fig. 4 illustrates the differences in the DSC values before and after the correction of the two plans mentioned. Analysis of the image registration of the CT, MRI, and PET datasets revealed no clinically relevant deviations.

**Table 1:** Volume [cc] of PTV60 (standard plan), GTVu, PTV60ex and PTV75 (experimental plan). Furthermore, the volume [cc] receiving 40 Gy and 45 Gy for standard and experimental plans. In brackets are the results after the correction in the second round.

**Table 2:** DICE Score, Hausdorff Distance (HD) and 95th percentile auf Hausdorff Distance (HD_95_) for the target volumes and OAR.

### 3.2 Dose comparison

All standard plans met the criteria for the median dose (range) covered by 98 % (D98, near minimal), 2 % (D2, near maximal), and 50 % (D50, mean dose) of PTV60. For the experimental plans, the median (range) D98 values of GTVu, D98 values of PTV60ex, and D98, D2, and D50 values of PTV75 all fell within the manual's requirements. Table 3 lists these values for each plan. The median volumes for V40 and V45 (volume receiving at least 40 or 45 Gy) for the Brain-GTV were measured at 302.8 cc (range: 211.3–411.5 cc) and 284.6 cc (range: 209.2–360.7 cc) in the standard plans. In contrast, the experimental plans exhibited V40 and V45 volumes of 284.6 cc (range: 209.2–360.7 cc) and 242.3 cc (range: 187.4–313.0 cc), respectively.

**Table 3:** The dose values of the target volumes for both plans of each participating center: the green values are completely within protocol; the yellow values are acceptable variations. In brackets are the results after the correction in the second round *(D98, D50, D2 = the dose covering 98 %, 50 % and 2 % of the volume standing for the near minimal, mean and near maximal dose, respectively; GTVu = the union of the gross tumor volume and the biological tumor volume; PTV60, PTV60ex = the planning target volume prescribed with 60.0 Gy of the reference plan and of the experimental plan; PTV75 = the planning target volume prescribed with 75.0 Gy of the experimental plan).*

### 3.3 OAR dose comparison

**Fig. 5:**
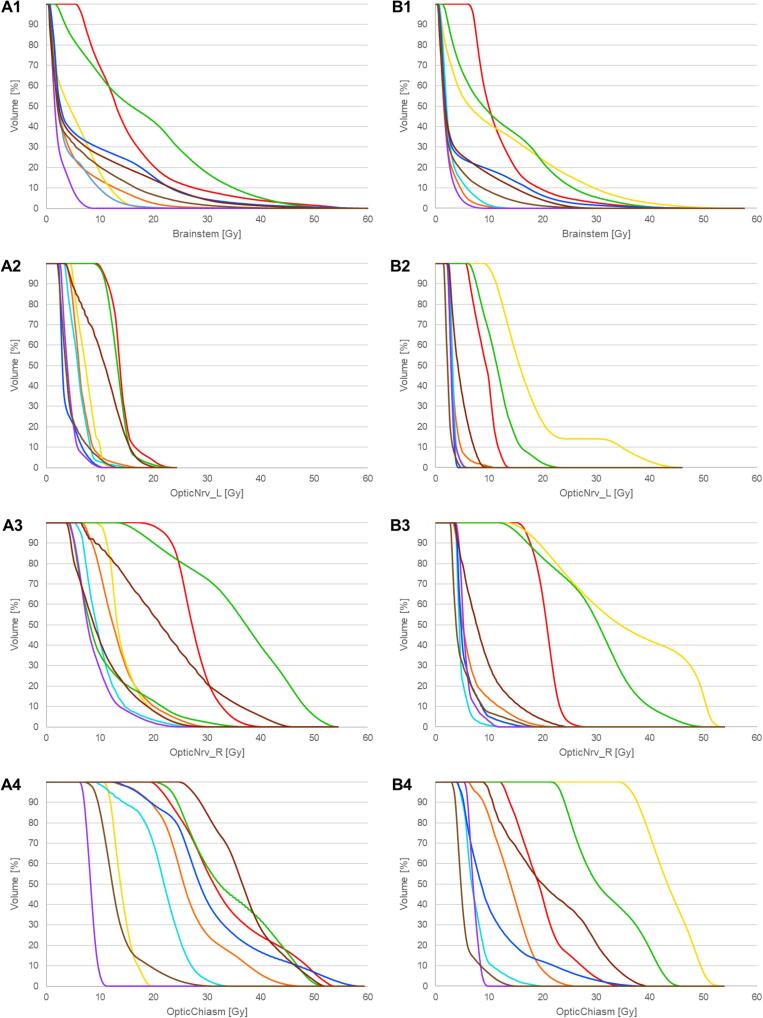
The dose-volume histograms (DVH) of the brainstem (A1, B1), the optic nerves (A2–3, B2–3) and the optic chiasm (A4, B4); A1–4 are from the standard plan, B1–4 from the experimental plan.

The exposure of the OAR also differed to some extent. For example, the median (range) brainstem exposure (D0.03 cc) was 41.9 Gy (8.5–57.8 Gy) for the standard plans and 29.8 Gy (8.6–52.9 Gy) for the experimental plans. Two standard plans ( red 56.0 Gy;  blue 57.8 Gy) had brainstem dose levels in the critical range according to the PRIDE constraints [1], [28]. For the brainstem center (brainstem minus 3 mm inner margin) the median D0.03 values were 26.4 Gy (6.7–47.8 Gy) for the standard and 21.0 Gy (4.7–42.6 Gy) for the experimental plans, respectively. The exact values for each patient and plan of the critical OARs are listed in Table 4. The corresponding dose-volume histograms are shown in Fig. 5. Dose values for all OARs are listed in the Supplementary Table.

**Table 4:** The dose exposure of the organs at risk (OAR): the green values are completely within protocol, the orange values are above or below the margin of acceptance, the non-colored values do not have any specific constraints *(D0.03 cc = the dose covering 0.03 cc; Dmean = the mean dose received by the volume; Ref = the reference plan; Exp = the experimental plan).*

### 3.4 EUD/NTCP

**Fig. 6:**
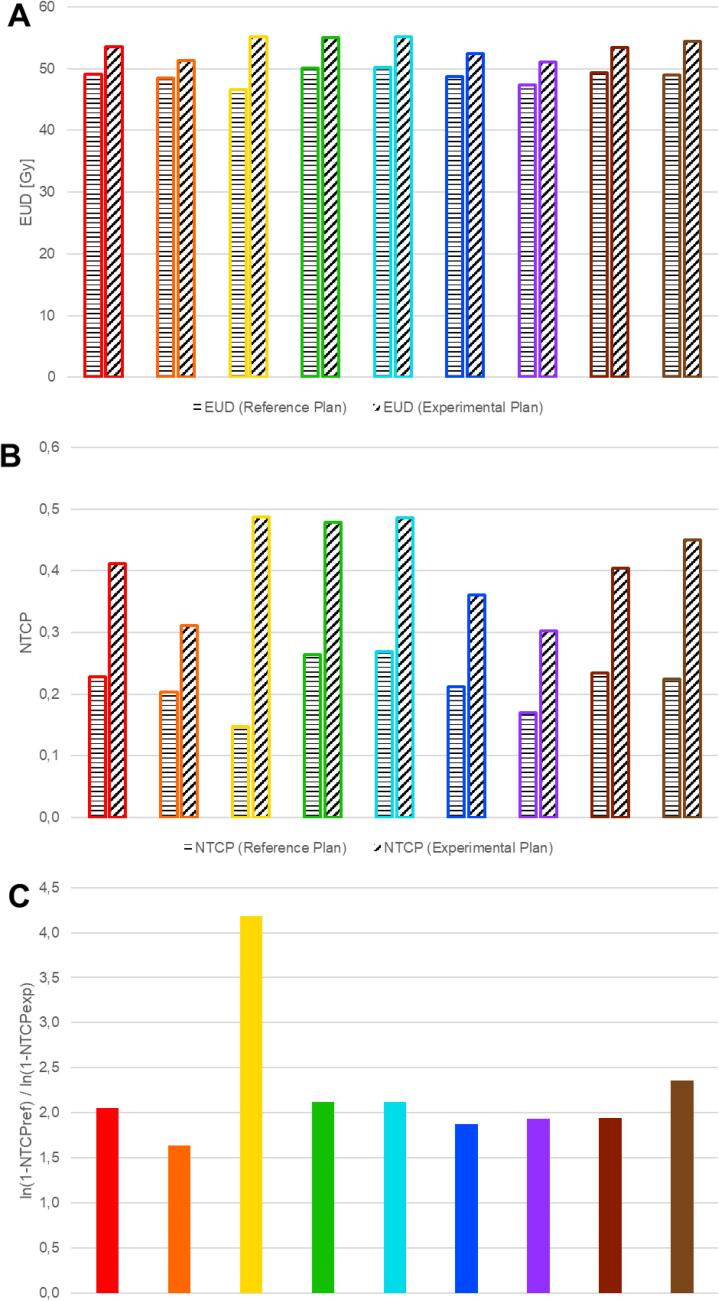
Diagrams depicting the EUD (A) and NTCP (B) values of each submitted plan in the standard and experimental plan. The third diagram (C) shows the results of a logarithmical comparison (EUD = equivalent uniform dose; NTCP = normal tissue complication probability).

The median (range) EUD of the brain was 49.0 Gy (46.6–50.2 Gy) and 53.6 Gy (51.1–55.2 Gy) for the standard and experimental plans, respectively. NTCPs were calculated as described: Median (range) NTCP_ref_ and NTCP_ex_ were 0.22 (0.15–0.27) and 0.41 (0.31–0.49), respectively. NTCP_ex_ was median 1.80 (range 1.53–3.30) times higher than NTCP_ref_. The logarithmic comparison showed a median value of 2.05 (range 1.64–4.19). With a NTCP ratio of 3.30 and a logarithmic comparison of 4.19, the yellow plan stood out. The exact values for each center, including the comparisons, are shown in Table 5, and a graphical representation of the EUD and NTCP for both plans is shown in Fig. 6.

**Table 5:** The parameters related with the risk of radiation necrosis; in brackets are the results after the correction in the second round *(EUD = equivalent uniform dose; NTCP = normal tissue complication probability; Ref = the reference plan; Exp = the experimental plan).*

## 4 Discussion

**Fig. 7:**
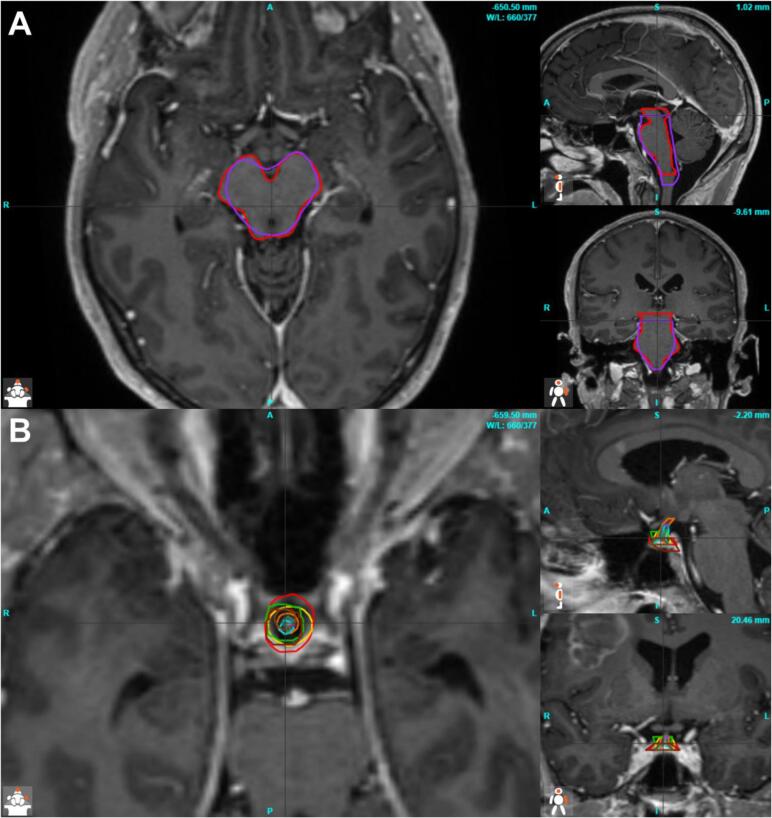
Exemplary comparative sections of organs at risk (OAR) to illustrate the differences. A: Brainstem comparison of one center (purple) to reference (red) B: Pituitary gland of all centers. (*A = anterior; P = posterior; S = superior; I = inferior; L = left; R = right).* (For interpretation of the references to colour in this figure legend, the reader is referred to the web version of this article.)

The deviations in the contoured structures and the final plans illustrate the usefulness of the performed dummy run. By providing targeted feedback to the sharing centers, all plans were approved in the end and the misunderstandings were clarified. At the same time, the dummy run revealed inaccuracies and shortcomings in the RT manual, which could be corrected before the start of the study. It also revealed technical challenges in uploading the plans into ProKnow®, which were addressed in collaboration with the local information technology (IT) and privacy officers. Once again, this demonstrates the need for a functioning QA system that is in place before the beginning of patient accrual. An example of a notable difference is the diminished brainstem exposure observed in the purple plan (8.6 Gy, experimental plan). This is attributable to the termination of the OAR structure two layers (6 mm) more caudal than the gold standard plan, and therefore exhibits a low brainstem DSC of 0.78 (Fig. 7A).

The goal of the PRIDE trial is to minimize radiation exposure to potentially unaffected brain tissue while intensifying treatment to the actual tumor volume for a more aggressive approach. The reduction of margins may provide additional benefits to OARs by allowing for smaller CTV-margins despite the dose escalation: By reviewing the dose values in Table 4 and examining the dose-volume histogram (DVH) plots in Fig. 5, it is clear that the critical OARs are exposed to less radiation in the majority of experimental plans compared to the corresponding standard plans. At the same time, the NTCP for the experimental plans is comparatively higher (Fig. 6, Table 5), but below the factor of 2 up to which we assume the efficacy of the protective effect of BEV, which can potentially halve the radionecrosis risk [7]. The difference of IR is greatest for plans of the yellow center, as the standard plan has the lowest NTCP (0.15) and the experimental plan has the highest NTCP (0.49). This is primarily attributed to the significantly disparate exposure of normal brain tissue (Brain-GTV) in the two center plans. The V40 for the standard plan, at 211.3 cc, is the lowest among all centers, while for the experimental plan, it is the third-highest at 327.5 cc. This pattern is reiterated in the V45 values, with 185.3 cc (lowest) compared to 283.3 cc (third-highest) for the standard and experimental plans, respectively. This is ultimately due to an incorrect transmission of the BTV structures and therefore also without any consequences for the actual study.

Margin reduction and dose escalation require the most accurate planning possible, as has been demonstrated in previous dose escalation studies [2], [3], [4], [5], [11]. For this reason, the dummy run plans were reviewed in great detail. Two plans incorporated edema, leading to a significant increase in the GTVu and PTV volumes. Consequently, EUD and V40/V45 values were also increased. The correction resulted in a reduction of the GTV volume from 102.1 to 50.3 cc, and from 84.1 to 53.3 cc, while the volume of PTV75 decreased from 142.7 to 78.2 cc and from 140.1 to 89.3 cc for both plans, respectively. This is exemplified by the DSC (Fig. 2; A1). The inclusion of the FLAIR changes has been partially recommended, and this misunderstanding was based on an ambiguity of the manual. Additionally, properly defining the exact FET-positive area poses a challenge. The recently published ESTRO-EANO guidelines [13] and PET RANO 1.0 criteria [29] provide further insights in this regard. As part of further analysis of FET-PETs, we have decided to use the 1.6 threshold for the trial in the future instead of the 1.8 TBR mentioned and used here. In the case of very small structures, such as the pituitary, which appear in only a few image slices, the limitations of geometric plan comparisons become apparent, but minor inaccuracies may still be detected.

ProKnow® has been used to compare the radiation plans of different radiation oncologists for a patient case in prostate cancer and bone metastases [30], [31]. In addition, it has been utilized to compare and analyze AI generated plans [32]. The National Health Service (NHS) currently uses ProKnow® to improve quality and reduce variability in radiotherapy services. A major advantage of this software is the high degree of compatibility between vendor-specific, proprietary software.

The results of the dummy run indicate that the PRIDE study design is able to meet its constraints and achieve adequate dose coverage and it showed the necessity of a functioning QA system. Furthermore, the increase in radionecrosis risk estimated by the logarithmic NTCP (IR) comparison is within acceptable limits. Therefore, if the hypothesis, that the risk of radionecrosis is reduced by BEV by the same factor as it is increased by dose escalation, is correct, isotoxicity may be achievable in the PRIDE trial. At the same time, familiarity with the manual and the planned conduct of the study was achieved and the technical requirements of the upload in ProKnow® were established. Nevertheless, there were still some relevant deviations with regard to the target volume definition; corresponding feedback was given to the participating study centers. All requirements were met after the subsequent correction.

## 5 Conclusion

The presented data illustrates the need for training prior to inclusion of the first patients regarding patient safety, quality management and protocol-compliant conduct of the study. A continuous upload of the radiation plans is integrated in the protocol, the first three plans of a center have to be approved by the leading study center before the start of therapy.

## Ethics approval and consent to participate

This study was approved by the local ethics committee of the Ludwig-Maximilians-University Munich (application number: 23-0068 fed).

## Consent for publication

Not applicable due to the retrospective nature of the analysis and the use of a non-identifiable sample case.

## Availability of data and materials

The datasets used and analyzed during the current study are available from the corresponding author on reasonable request.

## Funding

This project has received funding from the Bavarian Cancer Research Center (BZKF) and from Deutsche Krebshilfe (Project 70114648). Adrien Holzgreve received personal funding by the Bavarian Cancer Research Center (BZKF). We acknowledge support from the Open Access Publication Fund of the University of Tübingen.

## CRediT authorship contribution statement

**Sebastian H. Maier:** Formal analysis, Investigation, Writing – original draft, Visualization. **Stephan Schönecker:** Formal analysis, Investigation, Writing – original draft. **Vasiliki Anagnostatou:** Software, Visualization, Writing – review & editing. **Sylvia Garny:** Investigation, Writing – review & editing. **Alexander Nitschmann:** Investigation, Writing – review & editing. **Daniel F. Fleischmann:** Investigation, Writing – review & editing. **Marcel Büttner:** Writing – review & editing. **David Kaul:** Investigation, Writing – review & editing. **Detlef Imhoff:** Investigation, Writing – review & editing. **Emmanouil Fokas:** Investigation, Writing – review & editing. **Clemens Seidel:** Investigation, Writing – review & editing. **Peter Hau:** Investigation, Writing – review & editing. **Oliver Kölbl:** Investigation, Writing – review & editing. **Ilinca Popp:** Investigation, Writing – review & editing. **Anca-Ligia Grosu:** Investigation, Writing – review & editing. **Jan Haussmann:** Investigation, Writing – review & editing. **Wilfried Budach:** Investigation, Writing – review & editing. **Eren Celik:** Investigation, Writing – review & editing. **Klaus-Henning Kahl:** Investigation, Writing – review & editing. **Elgin Hoffmann:** Investigation, Writing – review & editing. **Ghazaleh Tabatabai:** Investigation, Writing – review & editing. **Frank Paulsen:** Investigation, Writing – review & editing. **Adrien Holzgreve:** Resources, Writing – review & editing. **Nathalie L. Albert:** Resources, Writing – review & editing. **Ulrich Mansmann:** Validation, Writing – review & editing. **Stefanie Corradini:** Writing – review & editing. **Claus Belka:** Resources, Supervision. **Maximilian Niyazi:** Conceptualization, Methodology, Validation, Writing – review & editing, Supervision, Project administration, Funding acquisition. **Raphael Bodensohn:** Formal analysis, Investigation, Writing – original draft, Conceptualization, Methodology, Validation, Writing – review & editing.

## Declaration of competing interest

The authors declare that they have no known competing financial interests or personal relationships that could have appeared to influence the work reported in this paper.

## Appendix A Supplementary data

Supplementary Data 1 Table S1: Reference Plan. The dose values of the target volumes and organs at risk for standard plans of every site: the green values are completely within protocol, the yellow values are acceptable variations, the orange values are above or below the margin of acceptance (*D98, D50, D2 = the dose covering 98%, 50% and 2% of the volume standing for the near minimal, mean and near maximal dose, respectively; PTV60 = the planning target volume prescribed with 60.0 Gy of the reference plan*). Table S2: Experimental Plan. The dose values of the target volumes and organs at risk for experimental plans of every site: the green values are completely within protocol, the yellow values are acceptable variations, the orange values are above or below the margin of acceptance (*D98, D50, D2 = the dose covering 98%, 50% and 2% of the volume standing for the near minimal, mean and near maximal dose, respectively; GTVu = the union of the gross tumor volume and the biological tumor volume; PTV60ex = the planning target volume prescribed with 60 Gy of the experimental plan; PTV75 = the planning target volume prescribed with 75 Gy of the experimental plan*).

## Footnotes

2 After receiving the official approvals, the provisionally assigned ARO-2022–12 trial number was converted to the final trial number ARO-2024–01.

3 https://plastimatch.org.
