# Supplementary material for: Dummy run for planning of isotoxic dose-escalated radiation therapy for glioblastoma used in the PRIDE trial (NOA-28; ARO-2024-01; AG-NRO-06)
Source: Clin Transl Radiat Oncol. 2024 May 4;47:100790. doi: 10.1016/j.ctro.2024.100790 (PMC11101689; doi:10.1016/j.ctro.2024.100790)
Supplement: Supplementary Data 1 — Table S1: Reference Plan. The dose values of the target volumes and organs at risk for standard plans of every site: the green values are completely within protocol, the yellow values are acceptable variations, the orange values are above or below the margin of acceptance (D98, D50, D2 = the dose covering 98%, 50% and 2% of the volume standing for the near minimal, mean and near maximal dose, respectively; PTV60 = the planning target volume prescribed with 60.0 Gy of the reference plan). Table S2: Experimental Plan. The dose values of the target volumes and organs at risk for experimental plans of every site: the green values are completely within protocol, the yellow values are acceptable variations, the orange values are above or below the margin of acceptance (D98, D50, D2 = the dose covering 98%, 50% and 2% of the volume standing for the near minimal, mean and near maximal dose, respectively; GTVu = the union of the gross tumor volume and the biological tumor volume; PTV60ex = the planning target volume prescribed with 60 Gy of the experimental plan; PTV75 = the planning target volume prescribed with 75 Gy of the experimental plan). [file mmc1.pdf]

Table S1: Reference Plan

PTV60	D98 [Gy]	57.4	56.9	57.6	56.4	57.1	58.0	58.3	57.8	57.5
PTV60	D2 [Gy]	62.8	61.0	61.1	61.9	61.7	62.2	61.3	61.0	61.7
PTV60	D50 [Gy]	60.2	60.0	60.0	60.0	60.0	60.0	60.0	60.0	59.6
Skin	D0.03 [Gy]	53.3	46.9	50.3	53.1	52.5	47.7	52.3	51.7	55.8
Glnd_Lacrimal_L	D0.03 [Gy]	9.6	13.3	3.6	14.8	11.4	5.3	5.1	13.9	3.6
Glnd_Lacrimal_R	D0.03 [Gy]	40.5	22.6	20.7	26.6	16.8	20.7	19.7	16.1	35.7
Glnd_Lacrimal_L	Dmean [Gy]	7.5	7.8	2.7	11.4	6.3	2.5	3.1	9.0	2.4
Glnd_Lacrimal_R	Dmean [Gy]	24.0	17.3	12.8	17.4	9.0	12.5	8.9	10.6	14.7
Lens_L	D0.03 [Gy]	4.3	3.6	2.2	4.9	2.7	1.7	2.0	2.5	1.7
Lens_R	D0.03 [Gy]	4.1	4.8	2.8	6.4	2.7	2.6	3.0	2.8	3.0
OpticNrv_L	D0.03 [Gy]	20.6	12.3	10.4	18.6	8.8	8.3	7.4	16.9	10.8
OpticNrv_R	D0.03 [Gy]	35.8	25.5	21.3	51.1	17.5	26.8	16.8	40.2	23.2
OpticNrv_PRV_L	D0.03 [Gy]	27.6	22.7	14.1	27.3	23.2	18.0	15.5	25.0	14.4
OpticNrv_PRV_R	D0.03 [Gy]	50.5	41.9	38.0	56.6	46.6	48.0	44.5	52.8	43.6
OpticChiasm	D0.03 [Gy]	50.2	39.7	16.6	47.7	27.3	51.0	9.2	49.2	22.5
OpticChiasm_PRV	D0.03 [Gy]	55.4	53.4	21.6	54.2	50.4	61.1	15.9	53.4	39.1
Pituitary	D0.03 [Gy]	23.1	27.2	10.0	31.9	14.3	22.1	4.3	21.1	4.0
Brainstem	D0.03 [Gy]	56.0	34.6	19.1	53.4	24.2	57.8	8.5	50.3	41.9
Brainstem_Centre	D0.03 [Gy]	45.4	17.5	15.3	47.8	11.9	38.4	6.7	38.7	26.4
Cochlea_L	D0.03 [Gy]	6.9	1.3	3.4	1.9	1.4	1.3	1.0	1.2	0.9
Cochlea_R	D0.03 [Gy]	9.3	1.6	1.4	12.3	2.0	1.6	1.3	1.4	1.6
Cochlea_L	Dmean [Gy]	6.7	1.2	2.7	1.9	1.3	1.3	1.0	1.1	0.8
Cochlea_R	Dmean [Gy]	8.2	1.4	1.3	12.2	1.9	1.5	1.3	1.2	1.5
Eye_L	D0.03 [Gy]	13.2	12.0	6.4	14.8	29.1	6.2	7.7	11.9	5.0
Eye_R	D0.03 [Gy]	48.5	35.4	26.3	16.4	39.9	29.3	23.7	24.3	42.4
Brain-GTV	Dmean [Gy]	21.1	23.9	19.8	28.8	25.0	22.7	19.6	26.3	23.1
Brain-GTV	V40 [cc]	302.8	278.8	211.3	389.0	411.5	273.2	248.1	383.6	373.1
Brain-GTV	V45 [cc]	275.9	245.5	185.2	349.6	364.6	242.6	218.9	330.6	329.7

Table S2: Experimental Plan

GTVu	D98 [Gy]	73.8	73.6	73.6	72.1	73.9	73.6	73.8	73.6	74.0
PTV60ex	D98 [Gy]	56.6	57.1	57.5	57.7	57.8	58.2	57.5	59.0	58.6
PTV75	D98 [Gy]	71.3	69.2	71.2	70.1	71.4	72.4	71.3	71.6	71.7
PTV75	D2 [Gy]	78.6	76.3	76.7	76.2	76.7	77.0	76.6	76.8	78.1
PTV75	D50 [Gy]	74.9	74.9	75.0	75.0	75.0	75.1	75.0	75.0	75.6
Skin	D0.03 [Gy]	57.4	46.5	55.1	53.0	56.7	57.4	56.8	64.0	60.9
Glnd_Lacrimal_L	D0.03 [Gy]	6.0	11.3	14.3	15.0	6.7	4.1	3.8	7.6	3.3
Glnd_Lacrimal_R	D0.03 [Gy]	33.5	22.5	19.9	33.2	11.6	17.6	17.4	12.5	28.2
Glnd_Lacrimal_L	Dmean [Gy]	3.9	5.5	9.7	11.3	4.3	2.4	2.4	3.8	1.9
Glnd_Lacrimal_R	Dmean [Gy]	18.3	14.6	12.3	20.9	6.1	5.6	6.8	7.6	9.1
Lens_L	D0.03 [Gy]	4.3	2.2	3.7	4.2	2.1	1.7	1.9	1.4	1.4
Lens_R	D0.03 [Gy]	4.9	3.0	5.0	6.6	2.4	2.3	2.6	2.0	2.6
OpticNrv_L	D0.03 [Gy]	12.8	7.1	39.1	19.7	4.4	3.7	4.4	7.9	4.1
OpticNrv_R	D0.03 [Gy]	24.6	16.6	51.1	45.0	6.6	11.0	8.8	18.7	14.6
OpticNrv_PRV_L	D0.03 [Gy]	16.0	16.8	50.8	26.3	9.1	6.8	11.1	16.2	7.5
OpticNrv_PRV_R	D0.03 [Gy]	37.1	30.7	56.4	54.1	26.7	33.3	29.1	34.1	28.8
OpticChiasm	D0.03 [Gy]	29.0	20.6	49.1	42.2	10.9	26.3	8.0	35.7	10.5
OpticChiasm_PRV	D0.03 [Gy]	42.9	35.7	56.6	48.0	33.4	52.2	18.0	46.3	19.0
Pituitary	D0.03 [Gy]	17.3	12.4	39.9	28.6	4.7	4.4	3.9	5.5	2.5
Brainstem	D0.03 [Gy]	45.6	13.0	52.9	45.1	13.7	47.8	8.6	29.7	27.7
Brainstem_Centre	D0.03 [Gy]	31.6	5.6	42.6	38.0	7.6	28.6	4.6	21.0	15.3
Cochlea_L	D0.03 [Gy]	5.5	0.9	3.2	1.5	1.2	1.0	1.0	0.9	0.8
Cochlea_R	D0.03 [Gy]	7.0	1.2	3.1	13.1	1.8	1.3	1.3	1.1	1.3
Cochlea_L	Dmean [Gy]	5.1	0.8	2.9	1.5	1.2	1.0	0.9	0.8	0.8
Cochlea_R	Dmean [Gy]	6.6	1.0	2.7	12.9	1.7	1.3	1.2	1.0	1.2
Eye_L	D0.03 [Gy]	9.6	9.2	19.8	15.8	19.9	5.2	8.1	4.8	4.6
Eye_R	D0.03 [Gy]	36.1	31.3	32.0	17.4	37.4	24.1	22.4	14.7	39.2
Brain-GTV	Dmean [Gy]	19.1	21.5	26.3	27.8	23.3	20.9	19.1	24.7	21.2
Brain-GTV	V40 [cc]	209.2	225.7	327.5	332.8	360.6	246.9	222.3	323.8	284.6
Brain-GTV	V45 [cc]	187.4	194.2	283.3	291.6	313.0	213.1	189.8	271.1	242.3
